# Religious and Spiritual Aspects in the Construction of Identity Modelized as a Constellation

**DOI:** 10.1007/s12124-018-9436-8

**Published:** 2018-05-25

**Authors:** Pierre-Yves Brandt

**Affiliations:** Faculty of Theology and Sciences of Religions, Institute of Social Sciences of Religions, University of Lausanne, Quartier Unil-Chamberonne, Bâtiment Anthropole, CH - 1015 Lausanne, Switzerland

**Keywords:** Psychological identity, Multidimensional model, Religion, Spirituality

## Abstract

This paper makes a case for an integrative approach to the field of psychology of religion and spirituality. Rather than relying upon or choosing one approach or theory to the detriment of all others, one can identify the construction of psychological identity, modelized as a constellation, as one of several possible « points of connection » serving as work site for the convergence, synthesis and exchange among the vast and rich variety of concepts, measures, theories and methods extant in the field. This work of integration can stimulate, and enrich our perceptions of the multiple dimensions, levels and valences of religion and spirituality.

## Introduction

### Goal of this Paper

This paper is not intended to be a polished, final synthesis, but rather to be a proposition of a more tentative character. I would prefer to think of it as a stimulus for research in psychology in the field of religion and spirituality. It builds upon the text of the first chapter of the manual published recently by the American Psychological Association (APA), the *Handbook of psychology, religion and spirituality*. In this chapter, the scientific editors of the handbook, Kenneth Pargament, Annette Mahoney, Julie Exline, James Jones et Edward Shafranske express their editorial intentions for the project. In their words, they have sought not only to « capture the current state of the field » of the psychology of religion and spirituality, but « also hope to push the field forward by encouraging greater integration. » (Pargament et al. 2013, p.5).

In effect, as they state, « *Multiplicity* and *diversity* might be the terms that most accurately describe the current status of the psychology of religion and spirituality. No single paradigm dominates the field. » (Pargament et al. 2013, p.4–5). This multiplicity characterizes *equally well* both the religious aspect and the psychological aspect of the interdisciplinary task assigned to psychology of religion and spirituality. On the religious side, « no single definition of religion and spirituality has achieved acceptance by researchers and practitioners » (p.5). And on the psychological side, they state similarly that « no single methodological approach (…) does exist in the discipline » (p.5). They could have added that, on the theoretical level, as well as in psychology in general, « no single theory » constitutes a framework of consensual work.

In this context, Pargament and colleagues emphasize the need for « an overarching organizing vision » that they label « *integrative perspective* » (p.5). Aware of the richness of religious and spiritual life, they do not claim that the solution resides in the reduction of this diversity into a single theoretical framework or methodological unification. « To grasp the extraordinary breadth and depth of religion and spirituality », psychologists are obliged to call upon « multiple concepts and methods » (p.5). Nevertheless, « to create coherence and wholeness » in the field (of psychology of religion and spirituality), it is essential, Pargament and colleagues say, « that we identify points of connection and interaction, possibilities for convergence and synthesis, and unanticipated questions and challenges » (p.5).

In my opinion, the work done in the domain of the psychology of religious and spiritual coping responds to this preoccupation. It is a good example of a point of convergence between different psychological theories (attachment, psycho-dynamic, emotion, cognitive, personality, psychosocial, etc.), offering many possibilities of convergences between different theoretical currents and psychological applications and the methods with which they are associated.

Then, similar to how the Committee for the Oscars prepares its nominations for « Best Film », I would like to nominate a candidate that might serve as a liaison between the very different approaches to psychology of religion and spirituality; I would like to nominate *the construction of the psychological identity*.

### Plan of the Paper

For this, I will proceed in the following manner: Firstly, I will start by specifying what I call construction of psychological identity. After having briefly shown how the theme of identity is generally presented in the social and political sciences, I will distance myself from the work of these disciplines in order to propose a more developmental approach to the construction of the psychological identity. This approach integrates the advantages of different currents in psychology.

Secondly, I will show, in the light of the diversity of religious systems, how the theme of the construction of psychological identity permits a comparative and integral approach to these diverse systems.

Thirdly, having finished with the most theoretical portion, I will present some research conducted on the importance of religion and spirituality for patients suffering from schizophrenia which clearly demonstrate the diverse roles played by religion and spirituality in the construction of the identity. A five-dimensional model will be drawn, which approaches religion or spirituality (R/S) as a constellation (in the way stars form a constellation). It shows how the theme of the construction of psychological identity offers possibilities for integrating not only the diversity of religious systems but also for integrating the *diversity of forms of religiosities*, something that more directly concerns the individual sphere, and therefore the goals of research (and applied) psychology.

Then, the theme of identity transformations, condensed here in the term « conversion » will then permit, in the fourth section, to show the integrative possibilities of the theme of the construction of the psychological identity at the heart of field of the psychology of religion and spirituality: we will stress here not only the integrative potentialities of the theme in that which concerns the diversity of those religious and spiritual systems but also in that which concerns the diversity of psychological approaches. We will discuss here the question of the universals of psychology.

Finally, I will conclude by a summing up of reasons that plead in favor of the construction of psychological identity as a theme of research, among others, responding to the need for integration making itself felt in the field of the psychology of religion, including cultural diversity.

### The Concept of Personal Identity: Not on a Sociological or Political Level

Let us begin by clarifying the meaning given to the concept of identity in the context of this article. Identity is a concept largely discussed in sociology and political studies. Topics like nationalism, or labeling of deviant marginal groups (Schur 1971) show how identity is connected with boundaries production and used to distinguish outsiders from insiders, enemies from friends, foreigners from citizens, etc. (Lamont and Molnar 2002). Religion, like ethnicity or language can serve to strengthen the boundaries between groups or or even facilitate acceptance into an exclusive group. In times of insecurity, tendencies to identity withdrawal are common: speaking the same language, or having the same ancestors, for example, can easily be invoked to justify inclusion (e.g. « Jews, Christians and Muslims are all sons of Abraham ») or exclusion (e.g. « you don’t respect the same religious laws »). Having or not having the same religion can as well become identity markers in inclusion (« Catholic, Orthodox, Presbyterian, Baptist, etc.: that does not matter, we are all Christian ») or exclusion (« Authentic Romanian are orthodox ») processes.

For these reasons, « identity » or « identity construction » are generally negatively connoted in texts written by sociologists or political scientists (Brubaker 2001). In this paper, these expressions will not be used with negative connotations, because I will not adopt this sociological or political perspective, but a more neutral psychological one.

## The Construction of Psychological Identity

My goal is to illustrate some aspects of religion in individual identity construction, with a developmental and life-span perspective. I am not interested in how individuals are labelled by others, but by the reflexive relation to the self which is reworked all life long. I define here psychological identity as the manner by which the subject organizes himself in order to answer the question « who am I? ».

The reflexive relation to the self *structures* itself around the innermost, most intimate center of being that is implicated in attachment bonds. It enriches itself as it develops, from the first experiences of one’s own body, progressing through the diverse experiences of awareness of self (Rochat 2003). This sense of self consolidates itself during the first two years of life through interpersonal relations (Stern 1985). The identity solidifies in connection with affective, cognitive and psychosocial development. *This* identity that I want others to recognize and *that* identity that I attribute to myself differ from each other: the child learns to keep a secret, to lie, the child’s idea of humor develops. With adulthood the psychological identity is formed by articulating individual identity and collective identity (Brandt 1997); the subject experiences the interface between subjective identity (that which is attributed to myself) and objective identity (that which is attributed to me by the environment).

The innermost intimate self around which it has begun to organize the construction of the psychological identity is now protected by diverse strata of which the outermost consist of the most formal categories of social identifications, according to a system that can vary from one culture to another.

The psychological balance depends on the capacity to construct sufficient cohesion between the different strata of identity. The self-narrative plays an essential part in this process, and, religion can play a central role in self-narrative (Brandt et al. 2017). From a Piagetian developmental perspective, this construction results from the interaction of the subject with his or her environment. A coherent biographical narrative articulates personal experiences with symbolic materials present in the context: family narratives, city or state history, values, models of psychological processes, of what can be understood as individual self, etc.

*Clinical psychology* literature is full of case studies showing how difficulties in (re)constructing a coherent biographical narrative can trouble psychological development, not only affectively but also cognitively, in the ability to build social relationships, etc. *Social psychology* has established how important it is to belong to a group: being integrated in a group to feel « at home » with some others. Socialisation groups are especially determinant during infancy and adolescence. Socialisation is shaped by society organization: are you socialized for all aspects of your life in the same group, or is your social integration fragmented by belonging to multiple groups (Triandis et al. 1988)? *Attachment theory* has also shown the impact of relationships with primary caregivers on psychological development. Did a secure attachment lead to a socialisation similar to that of the primary caregivers or, on the contrary, might an insecure attachment have provoked a rejection of the parents’ meaning-system, and the search for a new one (Granqvist 2010)?

All these domains of psychology contribute to demonstrate the integrative role of identity construction in psychological development. They emphasize various aspects that are used psychologically in identity construction, and are also present in the cultural context. Each culture carries its own conception of the *self* and of the way in which one articulates individual and collective identity (Mead 1934). In a definite context, this cultural framework influences in a decisive way the manner in which a given person constructs his or her (psychological) identity. Culture is meant here in the sense of cultivation or cultivating processes: it is the milieu in which the individual, like a plant, is growing. This growth does not follow a wild path, but a domesticated process.

With that said, let us examine how religion or spirituality can contribute to the construction of the identity of the individual, to the psychological identity.

## (Psychological) Identity: Integrative Potential of this Topic Confronted with the Diversity of Religious Systems

In the endless debates between anthropology and psychology, the role attributed to cultural context is central. Where psychologists seek to discern and demonstrate the elements that are universal in the human race, anthropologists insist on the unique, incomparable (Detienne 2000) quality of each cultural construction of self. From this point on, it may be asked whether it is possible to identify anything as universal, once religion is considered. For *if* psychologists are looking for psychological modes of functioning that are valid for the whole human species – *but* there is nothing that can be considered universally relevant once we examine religion – are not the scientists who call themselves psychologists of religion in a paradox?

### Religion and Spirituality as Meaning-System

To answer this question, let us specify what we call religion, and its role from a psychological point of view. What follows does not pretend to give a general definition of religion, but only what is necessary from a psychological perspective in relation to identity construction. Pargament et al. (2013), after declaring that « no single definition of religion and spirituality has achieved acceptance by researchers and practitioners » (p.5) nevertheless attempt to propose a definition. They define spirituality as « the search for the sacred » (p.14) and religion as « the search for significance that occurs within the context of established institutions that are designed to facilitate spirituality » (p.15). These definitions are not acceptable in their entirety.

Let’s start with that with which we can agree, which is the importance given to the institutional dimension. This makes it possible to distinguish with Grom (1992, p.367) the collective level of religion (*Religion* in German) from the individual level of religiosity (*Religiosität*). Religion is a meaning-system (Park 2005) offering a global worldview shared collectively. So, contrary to what Boyer (1994) or D’Aquili and Newberg (1999) may suggest, *religion is not natural*. It is anchored in culture and results from a long-time collective construction. The emphasis on this aspect is recurrent in the sciences of religions. An emblematic definition of religion is that of Melford E. Spiro, which Jonathan Z. Smith (1998, p.281) says has « gained widespread assent among scholars of religion »: religion is « an institution consisting of culturally patterned interaction with culturally postulated superhuman beings » (Spiro 1966, p.96). Apart from the institutional dimension, Spiro bases his definition on the notion of « superhuman beings ». As a consequence, non theistic traditions are excluded: « Spiro, frankly admitted that perhaps ‘religion’ is not a category universal to all human cultures (…) [and] that some lexically-defined religions in which superhuman beings do not figure, such as Confucianism, should simply not be classified as religion. » (Wilson 1998, p.156). The same lack of universality emerges when Pargament et al. (2013) base their definition of religion on that of spirituality, which itself rests on that of sacred. This is where we part ways.

In fact, the attempt to define religion by means of the concept of sacred does not simplify the problem, but only complicates it. As Smith (2004, p.102–103) shows, the definition of the sacred contrasts two traditions of interpretation: The French one, characterized by « an essentially spatial and classificatory understanding of the sacred and the profane » (p.102) and the « Germanistic tradition (…) which saw the sacred (or the holy) as a positive religious force and reality. » (p.103) Smith continues: « This deep conflict between two long-standing and influential traditions of scholarship gave rise to attemps at mediation, most famously by Eliade, to some degree influenced by Caillois. The cost, however, which seemed (and seems) to me unacceptable, was scanting of the anthropological in defense of the ontological. This is, in fact, a debate at least as old as Hume, now rephrased under the influence of various neo-Kantianisms, as to whether the sacred is best understood as an expression or an experience, as a representation or a presence. I side, with the French, in affirming the first member of these two oppositional pairs. » (p.103).

Does renouncing an essentialist definition of religion based for example on the concept of « superhuman being » or « sacred » destroy the distinction between religion and culture? Spiro thinks so: « religion can be differentiated from other culturally constituted institutions by virtue only of its reference to superhuman beings » (p.98). One can agree with him in the case of a society characterized by a homogenous worldview. In this case, religion is part of the culture. In contrast, in societies marked by pluralism, religion differs from culture. Thus, in a contemporary society marked by Western culture, for example, several religions are simultaneously present. In other words, in a given culture, there may be religious pluralism. At the same time, a religion can be inculturated: Christianity (or even Catholicism) in Haiti is not identical to that of Italy or the Netherlands, to take only this example.

The same can be said of spirituality. La Cour et al. (2012) asked 514 adult Danes about their understanding of the word « spirituality », and conclude that « a common understanding of the concept ‘spirituality’ does not exist in a modern secular context such as that of Denmark. » (p.77). At the end of their article, they nevertheless suggest: « A coherent use of the term spirituality in future research might therefore comprise spirituality understood as a *context-bound experience of relatedness to a vertical transcendent reality*. With this in mind, we might suggest that only three of the six understandings of spirituality found in this study qualify as research themes. These are spirituality understood as New Age ideology, as integrated part of established religion, and as striving towards a vaguely defined higher reality, opposed to religion. Conversely, spirituality understood as positive human feelings or relations, as selfish attitudes or as common inspirations is not recommended as a coherent topic for research. » (p.80).

With that in mind, it is not necessary to consider spirituality outside the research field of psychology of religion. The emic distinction between spirituality and religion (**«**I am spiritual but not religious **»**) is not a sufficient reason for using this distinction on a theoretical level (etic). All the data produced by people saying that they are spiritual but not religious can be studied inside the research field of psychology of religion. In this reason, spirituality is included in this field, because what people call spirituality can be described with the tools used for studying religion from a psychological viewpoint. Like religion, spirituality is a meaning-system (Park 2005) offering a global worldview connected to a « beyond » of the psychological experience of this world.

### Conceiving of a « beyond » Is a Psychological Experience

For its part, psychological experience of this world is perceptual, sensitive, cognitive, social, etc. In all these dimensions, skills can be measured; scales, scores, categorizations can be established, etc. However, skills, scores, categorizations, etc., are always relative. In each dimension, a maximum of what human beings can experience marks a limit(ation). We cannot feel under a certain level of intensity or above it, but we can conceive of a *beyond* what we can feel, hear, imagine, conceive of, etc.

To conceive of this *beyond*, and of its existence, remains a psychological experience. But the object to which one connects that experience is not in the field of psychology, because it cannot be grasped by psychological concepts or theories. Religions and theological discourses as well as non theistic spiritual discourses, on the contrary, pretend to grasp it. The experience of this *beyond* is called « spiritual » in the definition of La Cour et al. (2012) of spirituality as « a *context-bound experience of relatedness to a vertical transcendent reality* » (p.80). For some people, the vertical transcendent relatity refers to gods or superhuman beings. For others, it refers to inworldly powers that transcend the personal psychological experience (e.g. transpersonal experience). For others, it is the experience of autotranscendence. All of these understandings include the « possibility of another reality than the already known. » (La Cour et al. 2012, p.79). Being in relation with this other reality refers to what we describe when we use the word *beyond*.

### R/S Systems Claim to Give a Global Interpretation

Religions as well as various forms of spiritualities, as global worldviews, *claim* to propose meaning-systems that integrate all aspects of the life of a human being. They offer references for constructing a coherent biographical narrative, including a community of belonging, attachment figures on which attachment bond can be transposed, values, models and roles, etc. These references are provided by texts and discourses, practices and rituals, and also by people playing roles in definite institutional structures.

Nevertheless, as we have seen, the sciences of religions will stress the uniqueness and incommensurate quality of religious systems. There are no universal features common to all religious systems, despite various attempts to postulate a common core in one (specific) psychological experience. Among these attempts, we might list conversion as an endogenous process (Hall 1904), the father complex (Freud 1913), the experience of the ego transcended by the Self (Jung 1938), the « numinous » experience (Otto 1917), the need for a secure attachment (Kirkpatrick 2005), the mystical experience (d’Aquili and Newberg 1999), etc.

And yet, that which we call religion, and all that we put under that heading, ought to have something in common. The problem is that there is no constitutive feature (gods, the sacred, etc.) common to all religions. However, absence of universal common features does not mean having nothing in common. There is no universal, essential feature common to all religious and spiritual systems, but religious and spiritual systems all claim to fulfill the same function in society. This common element, I define as the claim to give a global interpretation, that is to say encompassing the entirety of human activity to the question of the meaning of life.

### R/S Systems Have in Common the Question of Identity

Fundamentally, that which all human societies have, especially through their religious productions, in common, is that they suggest answers to the following questions: **«** Where do we come from, why are we here, where are we headed? **»** Their answers are diverse, but the questions are common. The religions have in common some existential questions to which they try to answer in incorporating the entirety of human life, even the entirety of life in the world. They have, each of them, potentially, a claim to be the one bringing a universal answer, because they attempt to respond to universal questions. On the individual scale these questions could be expressed thusly: « Where do I come from? Why am I here? Where am I going (to)? » From a psychological point of view, they can be resumed in the question: « Who am I? » It is the question of (individual) identity.

It is not only R/S that provides answers to these questions. Law, history, science, etc., address these questions as well. A claim of religions, and also, in large part, of the various forms of spirituality that distance themselves from traditional religions, is that they provide a basis for these responses which can only be partial. Each R/S system treats this question differently. The diversity of responses to this question is an expression of the diversity of R/S systems. Consider just a few examples from some religious traditions. In the Torah, fundamental source of Judaism, the response to the question of identity is even a way of characterizing the divine. « I am that I am **»** or « I am who I am », « I will be he who I will be **»** or « I will be what I will be » are attempts to translate the response that God gives to Moses who asks who he is (Book of Exodus 3:14). In the whole of the Jewish tradition, the identity of the individual is inseparable from being part of the people who belong to *this* God (« You are my people », Book of Exodus 6:7; Book of Jeremiah 30:22), to the point where, from the period of rabbinic Judaism, it stabilises the rule that the Jewish individual identity is transmitted by the mother, making the conversion to Judaism very difficult.

Identity is also a central topic in the Christian Scriptures. The question of the identity of Jesus is found throughout the Gospel texts, the essential part of the canon of Christian Scripture (in the New Testament). Jesus himself asks the question of his disciples: « Who do you say that I am? » (e.g. Gospel of Mark 8:27). These writings try to answer the question « who is He? ». The answer to this question developed in the other texts of the New Testament consists of the title « Son of God ». This divine filiation is in turn transferred to those who, embracing the Christian faith, become brothers and sisters of Jesus (e.g. Epistle to the Romans 8:29).

In Islam, *muslim* means one who surrenders and submit his or her will to Allah. The name of this religion itself emphasizes that which constitutes the nucleus of the identity of the member of that religion. It is an answer to the question « Who am I? », « Who are we? ». In India, the Hindu concept of *atman* refers to the eternal and real self, beyond the ego. It is another way to answer the question « Who am I? ». In Buddhism the true identity is that of the Buddha, the Awakened One (the Enlightened One). The Buddhist path consists of escaping from the illusion of false identities resulting from one’s attachment to the ego. By this renouncement one seeks to attain *enlightenment*, which is to become the real being lying dormant at the center of oneself. These few examples illustrate the centrality of the theme of identity in religious systems.

This question of the identity constitutes, in my opinion, a major object of study for the psychology of religion. The study of this object consists of posing and answering the question: How does religion provide the resources for answering the fundamental (psychological) question « Who am I? **»**. We have tried to show how the question of identity, because it is a question common to diverse religious systems, constitutes a privileged point of connection between them. This question offers thus a structure of study especially apt for an integrative approach to religions. We can now see how this thematic offers, as well, the possibility of an integration of diverse dimensions of the religions, and thus the variety of forms of religiosities.

## (Psychological) Identity: Integrative Potential of this Topic Confronted by the Various Forms of Religiosities

To begin with, let us note that, from a psychological point of view, being able to integrate a variety of forms of religiosities is more important than the ability to integrate a variety of religious systems. In effect, the psychology of religion can access religious systems only through the study of human subjects who refer to these systems, thus only through the religiosities of these subjects. Thus, it is *most importantly by individuals manifesting this* variety of forms of religiosities that the religious diversity comes to the psychologist. This variety of forms of religiosities (including a variety of forms of spirituality) is a result of the multidimensional character of religion and of spirituality. What is more, it is also a result of the variety of religious systems accessible to individuals.

### Multidimensionality of Religion

We understand today that religion is not just having beliefs or practices. Religion is multidimensional, as is, by the way, spirituality. In the first chapter of the *Handbook of Psychology, Religion, and Spirituality,* Pargament et al. (2013) refer to the models of Glock (1962) and of Idler et colleagues (2003). Both reflect the multidimensionality of religion. Consequently, each individual has the freedom to reflect *differently* on the importance that he accords to diverse dimensions of the religious system(s) to which he refers. The result of this reflection is marked notably by the manner of exploiting religious or spiritual resources originating from one or more religious systems in the construction of one’s (psychological) identity.

### Multidimensionality of R/S Often Ignored in Research: The Case of Religion and Health

Unfortunately, this multidimensionality is not always appreciated to its proper extent. The research carried out in the broad field of studies on the links between health and religion is a good illustration. It suffices to consult the handbook edited by Koenig and colleagues on religion and health (Koenig et al. 2012) to remark that many studies reduce the measure of religiousness or spirituality to one or two (more sociological) questions: religious affiliation, frequency of collective practice, or frequency of individual practice. Of course, in many cases, the diversity of forms of religiosity and of spirituality have simply not been taken into account because it would have required too long an investigation given the state of health of the patient or the time available for study. However, this is more often a result of a lack of theoretical knowledge about what constitutes religion and spirituality. Too few have sufficiently studied the domain to be able to understand that there are forms of religiosities quite diverse. For certain of these forms of religiosities, none of the measures proposed would be pertinent. For some people, religion means above all to follow ethical principles. For others, it is mainly to meet food or sanitary requirements (diet), and this sometimes may be without attending a religious community, without religious practices of prayer, or without collective or individual ritual practices.

### Importance to Approach R/S Multidimensionally Is Illustrated by our Research on Schizophrenia

The research on patients suffering from schizophrenia that we conducted in Switzerland and Canada with colleagues (Brandt et al. 2012) will help to illustrate how important it is to approach religion really multidimensionally. We are going to show how a model that integrates five dimensions of religion can serve as a theoretical framework for an integrative approach to religious multi-dimensionality for religious coping, and how its use can be extended to the construction of the (psychological) identity.

The research was begun with the doctoral thesis of Sylvia Mohr (Mohr et al. 2006) upon a population of about a hundred patients suffering from schizophrenia in Geneva. These patients were outpatients with chronic psychosis, aged between 18 and 65 years. The objective was to discover to what degree religion and spirituality helped them to deal with their illness. Religion and spirituality were assessed through a semi-structured interview. The same research was replicated in Trois-Rivières (Quebec, Canada). Results show that religion is considered as moderately important or essential by around two-thirds of the patients in Geneva (Switzerland) as well as in Trois-Rivières, and helpful in making sense of life and illness (Borras et al. 2010).

After this first (more fundamental research) stage, a second stage took a more strongly applied direction. The goal was to build a model of intervention (Huguelet et al. 2011). We wanted to test how the results of our first research stage might be applied by practitioners. We wanted to see to what measure it would be possible to ameliorate the treatment of patients suffering from schizophrenia taking into account the results of the research on the importance of religion and spirituality. The idea was to teach the consulting psychiatrists of the outpatient walk-in clinic how to use our religious and spiritual assessment (Mohr et al. 2007; appendix) in order to be able to identify some aspects of the patient’s religiosity or spirituality that could be used as a theme in the treatment in order to effect a better recovery. We had in view the possible mobilization of religious or spiritual resources that encourage the patient to rely upon said resources in order to cope with their difficulties. It was also envisaged that the practitioner could encourage the patient to meet with a pastor, chaplain, etc. in case of negative religious coping. We also wanted to open the possibility of working on patients’ representations of psychiatric disorder and treatment. For some patients, the treatment is experienced as antagonistic to religion or spirituality. Working on the nature and causes of psychiatric disorder and how the treatment works seems necessary when religious meanings given to them interfere with understanding why the treatment is prescribed (Brandt et al. 2012, p.200).

The psychiatrists attended a ninety-minute training session during which they were introduced to the method of conducting the religious and spiritual assessment. This assessment served only to give guidelines to permit the psychiatrist to note whether religion or spirituality had any importance for the patient and, if so, in what way. Thus there was no obligation for the clinician to investigate each topic in detail. Rather, the idea was to give them a sufficiently large perspective on different aspects of religion or spirituality that could be incorporated into the coping. For example, if the patient said that he never frequented religious groups, and that he was helped by non-religious meditation, the psychiatrist would be inclined to leave the other aspects of religion and spirituality and concentrate the session on that which the patient considered to be worthwhile. In effect, the interview took place in the course of regular consultations with the patient (on an average of once a month for about thirty minutes), where the psychiatrist, when suggesting to take time to discuss religion or spirituality during the interview, had to be careful to remain patient-centered.

After the initial assessment, the participating psychiatrists received supervisory sessions with a psychiatrist, and a psychologist of religion. During these individual meetings, the clinicians reported the outcome of the spiritual assessment they had conducted. For each patient, the participating psychiatrist was given guidance and advice, similar to clinical coaching. Suggestions for treatment discussed in these meetings were then categorized by four judges as part of a consensus process. This resulted in six themes: support of existing positive religious coping or work on religious struggle, mobilization toward clergy or religious community, work on identity or values, differentiating delusion from faith, work on patient’s representations of psychiatric disorders and treatment, and no intervention related to religion/spirituality. Three months later, the clinicians were invited to report their evaluation of the intervention (Huguelet et al. 2011, p.83; Brandt et al. 2012, p.199).

From this supervision work, we can conclude: *Too often religion is confused with beliefs or with mere rituals*. While clinicians might recognise that religion can help patients cope with their illness, they often have a narrow understanding of what religion means, reducing it to a mere stereotypic representation. In fact, the clinicians reported at the end of the study that the ninety-minute training session was insufficient to familiarize them with the domain. One stereotype about the collective side of religion can be expressed by the phrase « religious people attend church **»**. So, a patient who declares an interest in attending church will be encouraged to do so. Another stereotype relates to the individual side of religion and is summarized by the phrase « spiritual people pray or meditate alone at home **»**. Patients who declare such individual practices are likely to be encouraged to persevere in these practices.

There is no doubt that some strategies of religious coping follow these stereotypes; however, other strategies of religious coping are often underestimated or simply unrecognized and ignored. The case of a Muslim patient (Brandt et al. 2009, p.169–170), Mr. Z., who clearly confirmed that getting back in touch with Islam helped him face his difficulties with illness, illustrates also that religious coping is not only made by religious beliefs or practices (e.g. prayer, meditation, attending services, etc). The current problem of Mr. Z. was to endorse a father model for his children, but the practitioner was struggling to consider resources provided by religion. Until the moment when, thanks to the supervision work, the psychiatrist was able to realize that a parental role can also be modeled by religious referents.

In this case, the construction of the psychological identity was enabled by the ability to assume a role as parent and even, more precisely, the paternal role of the family. And it is through work on this identity construction that Mr. Z. can effectuate a better coping with the difficulties experienced, and to know how to improve his self-esteem. This case illustrates that there are several ways in which religion or spirituality can be seen to influence the process of recovery.

### R/S as a Constellation: A 5 Dimensions Model

The categorization of treatment suggestions in six themes was made from the perspective of medical intervention. The purpose of this categorization was to better understand when and how R/S can be considered as a resource on which it might be beneficial for the patient to work on. After these initial analyses, the same set of suggestions was reworked from the perspective of multidimensionality of R/S. As a result, it has been possible to identify five aspects (Brandt et al. 2012), which can be described as a constellation:

- *Community aspect*: The need for social integration and support (1) leads to participation in collective religious activities.
- *Intimate relationship*: The need for security and protection (2) leads to the construction of an intimate relationship with a « spiritual figure », no matter whether it be with a supernatural agent (deity, saint, etc.) or a spiritual guide (priest, spiritual counsellor, etc.) understood as a mediator with spiritual realities.
- *Rules system*: The need to hold endogenous impulses (3) leads to a search for (and a defense of) religious systems of rules, rites, and practices.
- *Identity construction*: The need for identity and self-esteem (4) leads to an identification with religious roles, models, and narratives.
- *The world view aspect*: Finally, the need to implement a meaning system (5) looks for support and help in reframing one’s world view (differentiating delusion from faith, reshaping representations of illness, treatment, etc.).

How are these different dimensions organized in an individual? The way they develop or change over time depends from one individual to another. That is why, at the individual level, forms of religiosity can be very variable. For some people, one aspect may be much more important than all the others. Some people are mainly religious by affiliation, others by insertion in collective activities, others by adherence to a system of thoughts, when others by the respect of ethical principles or by individual practices and rituals. Others may still combine many or all of these aspects. However, such diverse people may very well all claim the same religious tradition. The fact that an individual moves from one form of religiosity to another depends very much on the circumstances he or she encounters during his or her life course, like meeting people with other ways of life or convictions, moving from one country to another, serious illnesses, etc. The case of the Muslim father who faces difficulties in endorsing a parental role shows how the growth of his children pushes him to move from a religiosity based on the system of rules aspect to a religiosity that also integrates the aspect of identity construction (identification with the father role as defined in Islam). Only the study of such practical cases can go into more detail in the possible reconfigurations of individual religiosity within a given society, but this is actually beyond the scope of this article.

At the moment of undertaking an applied research, it may not be necessary to use an extremely detailed measure of religiosity or of spirituality in order to guarantee that multidimensionality is taken into account. It might be more effective to conduct a *customised* research adapted to the patient or, more generally, to the target population. But this supposes having a model of this multidimensionality in the background, such as the model of five dimensions that I just presented.

It is also possible to assess the importance of religion or spirituality by simply beginning with a general question like: « Have you any religious beliefs or religious practices? Or do you have a spirituality? Or are you part of a religious group? ». Depending on what the answer may be, one will explore one or the other of the five components of our model, going deeper when necessary, then touching on the other components if necessary. This is a method that could, depending on the patients, lead to a settling of the question in several minutes, concerning those for whom religion or spirituality does not play a role, and open a more profound exchange which may enrich the interview underway, as well as several following consultations. But, in order to do this, it presupposes a knowledge of the model.

In our studies on religion and spirituality (R/S) in patients suffering from schizophrenia, we are centered on the integration of religious and spiritual resources for coping with the illness. In the model of R/S that we have drawn, the « construction of the identity » is one aspect among five. In this model, it is derived primarily from roles, models and narratives, notably narratives about oneself, coming from the religious or spiritual traditions aiming to integrate the diverse aspects of ones own identity in construction.

As an illustration of this dimension, we have taken the example of the father of the Islamic family. In this example, one can see very well that religion is not made up only of beliefs and practices/ritual and that religion concerns social roles as well. But we can go further with this idea. There are people who do not pose the question of belief or who do not believe (or who are not believers) and who consider themselves to have a religious identity simply because they have a religious affiliation. They observe, for example, certain rules of Judaic or Christian life, but are completely unable to answer if they believe in God because they have no personal relationship to Him and never pray. Their particular form of religiosity is not principally related to a belief in God nor to pious practices nor to specific social roles, but to a system of values that can be expressed in any social role and any life situation.

Thus if one is interested in the construction of the religious identity, one must consider how the other aspects of the R/S contribute to that construction: the aspects of socialisation in a religious community, the intimate relation with a spiritual figure, the system of values, the representation of the world.

### Identity Construction: A Constellation of five Components

Looking at things from the point of view of the construction of identity means that this aspect, non-dissociated from others, can also be seen as integrating the four others. Roles and models integrate the relation with a « belonging » group (if the collective dimension is of importance), a possible attachment relation with a spiritual figure, the respect of rules and principles, and the understanding of one’s own place in the vision of a coherent world. In other words, identity construction, like religion, can be described as a constellation of at least five components. Thus it integrates (Brandt 2013):

- The dimension of being in rapport with the community (the social aspect of identity construction, need for socialisation, social integration / the individual identity constructs itself in part by identifying with the group to which it belongs: I am a member of this religious community.)
- The dimension of attachment (the psychological stability of the identity depends on being able to have available a stable figure of attachment, which is not necessarily to say that attachment to this figure ought to be secure (!) / it is the dimension of the intimate relation: my spiritual figure of attachment is...)
- The dimension of rules and of values (ethical aspect of the identity: personal system of values, principles and rules for nutrition, sexuality, bioethics, male-female relationships, parent-infant relationships, etc.)
- The dimension of roles and of models (not the same thing as a collective identity: one can have the same collective or social identity as someone else, but have a role completely different in the society, the group, the family: my family roles are...; my identification models are...)
- The dimension of world vision (the history of the world to which I subscribe is...)

## (Psychological) Identity: Integrative Potential of this Topic Confronted by the Variety of Concepts, Measures, Theories and Methods Extant in Psychology

Now, how can it be integrated in a paradigm of the role of R/S in the construction of psychological identity?

### Modeling of Individual Courses of Identity Transformation(s)

The second area of research upon which I propose to reflect is that of the identity transformations organized around religious or spiritual references. This field of study is often referred to by the generic term « conversion » that we will adopt in order to simplify. We will use this term in the knowledge that it condenses a variety of identity transformations of spiritual or religious nature. It is not intended to refer only to those who have undergone the experience of being « born again ».

The study of religious or spiritual identity transformations is an important contribution to the elaboration of integrative models of psychological identity construction in connection with spiritual or religious points of reference. The identity transformations connected with the religious or the spiritual evoke several psychological theories.

### Modeling of Identity Construction: Integration of Psychological Theories

The modeling of identity construction that occurs by means of these transformations is therefore an excellent way to integrate different psychological theories. Thus, for example, the work on *religious attachment* permits one to think that the type of conversion chosen by a subject depends on the style of attachment built on experiences with primary caregivers. If we resume in a schematic way the hypotheses of Granqvist and Kirkpatrick (2004), those who had formed a secure attachment with their parents would tend to adopt the form of religiosity of their parents, according to a process that can be described as progressive conversion. In contrast, those who have put into place with their parents an insecure style of attachment, would tend to turn away from the religiosity of their parents and adhere to another form of religiosity in the manner of a rupture that is termed sudden conversion. Without going into the pertinence of these hypotheses, one can draw the conclusion that religious attachment can contribute to the construction of the individual identity, and thus that the theory of attachment contributes to the understanding of the process at work in this construction.

However, this theory is not the only one at work in the psychological construction of identity. *Social psychology* demonstrates with evidence the role played by the relation established between the convert and the group to which the convert belongs. The identity constructs itself in reference to a mode of socialisation, either by conforming to a dominant group, or in seeking refuge in a group that is marginal. The plurality of religious groups offers in our contemporary society various modes of affiliation and different paths of belonging. But the construction of the personal identity cannot escape the social reference, which likewise brings along with it a religious and spiritual dimension that is very much in evidence.

So far we have mentioned two psychological processes at work in the construction of the individual identity: the transposition of the need for attachment to new figures in the course of development and the need for socialisation to find one’s place in society. These two processes, which are both the objects of theoretical elaborations, could motivate access religious and spiritual resources. A spiritual figure can play the role of attachment figure, *and* belonging to a religious community can function as a central composite in the manner in which an individual builds his/her self, organizes his/her social identity, builds the representation of his/her self.

But these processes are not the only ones at work in the psychological construction of the identity. As a result, other religious and spiritual resources can be mobilised to take charge of other aspects of identity construction. Thus the process of identification at work in the course of *psycho-affective development* can apply itself to figures that are chosen for religious reasons. Certain conversions are, for example, caused or influenced by a romantic choice. The convert adopts the religion of his or her beloved. Or, the identification functions with a religious figure taken as a father-figure or mother-figure. Or, still, the convert finds in the religion to which he adheres an ideal of the self or the ego which helps him to construct his identity.

Identity can be delineated also in *cognitive* terms. It can find expression in the search for a vision of a coherent world, including a system of values. The religious conversion is often accompanied by an argumentation showing how this new vision of the world solves an interior conflict or some contradictions, or even a problem that seems up till now unsolvable. These modifications on the cognitive level would be theorized in the framework of cognitive-behavioral approaches or treated using problem-solving modeling.

One can observe that religious conversion – regarding it as being a kind of identity reorganization – touches different aspects of the person. It concerns in part disjointed (or non-articulated) psychological theories. One can look at conversion from the point of view of the theory of attachment, from a psychoanalytic, cognitive, moral, social point of view, etc. Taking up the task of considering what it might be in terms of (psychological) construction of the identity, one adopts a global perspective on the psychological development of the individual that favors an integration of diverse theoretical movements. This integration seems all the more necessary when one starts to be interested in some identity transformations linked with religion or spirituality. Because, as we have just seen, religion and spirituality furnish enough resources between them to be able to respond to diverse components of the construction of an identity that is not only individual but also collective.

In this sense, the construction of psychological identity seems very much like a viable « point of connection » between a variety of concepts, measures, theories and methods extent in psychology permitting the integration of multiple dimensions, levels and valences of religion and spirituality.

This integrative character of identity construction manifests itself particularly in self-narratives. Numerous works show that the construction of identity does not operate without narratives. This to the point where, in the framework of identity transformations as sudden (and totally reorganizing) conversions, certain authors judge that there is no conversion without a narrative of it (e.g. Harding 1987). It is through narrative that conversion operates. The status of a vocation narrative (or a confession of faults) could be considered in the same way. Thus, religious traditions provide accounts of identity transformations (vocations addressed to prophets, enlightenment of Prince Shakyamuni which leads him to leave the family palace, the conversion of Paul, etc.) These accounts can function as models to which anyone can identify (telling how an episode of his/her life conforms to the story of the Prophet, Abraham, Paul, the Buddha, etc.).

## Conclusion: Extensions

In the introduction to this paper, we cited Pargament and colleagues who talked of the need « to create coherence and wholeness » in the field (of psychology of religion and spirituality), in identifying « points of connection and interaction, possibilities for convergence and synthesis, and unanticipated questions and challenges » (p 5).

In my opinion, the work conducted by Pargament himself and by other colleagues who came after in the domain of psychology of religious and spiritual coping respond to this issue. It is a good example of a point of convergence between different psychological theories (attachment, psycho-dynamic, emotional, cognitive, personality, psychosocial, etc.), offering many possibilities for convergences between diverse theoretical movements and the methods associated with them.

The study of coping focuses on the individual in a state of stress. It examines how the person and his environment apply themselves to face their difficulties. Coping strategies are not without links with the construction of identity. Facing difficulties puts a strain on the construction of identity, that is to say the modalities of relationship with oneself and with others, and can cause identity rearrangements. But life is not made up solely of crises and struggles, and the identity does not form itself exclusively during these hard periods. A developmental approach ought to take into account, as well, the process at work outside of these moments. Intended to be seen as a part of the framework of psychology of religion and spirituality, the study of the construction of the (psychological) identity offers the possibility to construct a model for the integration of diverse dimensions of psychic life, while distancing itself from classic stages-models of religious development.

### Integration of the Diversity of Religious Systems

Answers given by religious traditions to the question of identity are diverse, but all traditions have the question of individual identity in common. This question opens a common research field for integrating the diversity of religious systems. In this research field, we can study how religions provide resources for answering the question « Who am I? » and try to integrate the results in a general model of the role of religion/spirituality (R/S) in the construction of psychological identity.

### Integration of the Multidimensionality of Religiosity

In studying the variety of forms of religiosity and spirituality in patients with schizophrenia, we were able to develop a Five-Aspects Model. Transposed to the topic of identity construction, it offers an integrative approach of the multidimensionality of R/S in the construction of psychological identity. In terms of integration of the multidimensionality of religion, the theme of the construction of psychological identity seems to open a productive approach.

### Integrative Character of Psychological Identity

Pargament and colleagues write: « We find religion and spirituality in every dimension of life. Theoretical and empirical studies clarify that religion and spirituality are multidimensional constructs, made up of a myriad of thoughts, feelings, actions, experiences, relationships, and physiological responses which serve many purposes and yield a number of consequences (e.g. Glock 1962; Idler et al. 2003) » (Pargament et al. 2013, p.5).

The construction of psychological identity integrates all these dimensions. Resources for this construction can be found in R/S. However, it becomes difficult to identify and examine anything universal when one is engaged in the psychology of religion. The only *things* that are universal when one looks at religion and spirituality are those existential questions to which religion and spirituality are trying to respond.

### Integration of a Diversity of Psychological Theories and Methods

Further along in their introduction to the Handbook, Pargament and colleagues describe the multi-dimensionality of the religious and the spiritual in human life. « Religious and spiritual pathways are constructed out of raw materials of cognition, affect, behavior, relationship, and biology » (Pargament et al. 2013, p.6).

These different components all stem from psychological theories and methods that are quite varied. We have mentioned some of them. The risk is that of a fragmentation of all that which can be expressed in psychology of religion and spirituality. Each theory can be used to justify itself or to clarify some religious or spiritual attitudes, comportment and experiences. The thematic of the role of religion and of spirituality and the construction of the (psychological) identity offers a fine opportunity to integrate both of them into a single model.

### A Renewed Way to Look at Development

This thematic permits to re-think development with an approach that avoids the model of stages, using instead integrations of components, probably, for certain aspects without a prescribed order; we could begin with a « stratified » model.

### Integration of Cultural Diversity

Finally, let us add that another important aspect, if we want to build integrative models, is the necessity to conduct intercultural research. In mentioning this field of research, I am not really interested in adding even more examples to illustrate the diversity of components integrating into the construction of the identity; we have sufficiently detailed that in the preceding parts. We just have to be conscious that the models described until now vary from one religious tradition to another, from one culture to another. It is the impact of the cultural referents that we must keep in mind. The identity construction of a subject depends on the cultural referents that are at his disposal. Then, intercultural comparison offers the possibility to distinguish between what is proper to a given culture and what is more general.

Such are the thoughts that I wanted share. They are not, such as they are, proven points, yet. It is as if I were inviting you to take a stroll with me through a garden of possibilities. The psychology of religion and spirituality is becoming more and more international, and studied more and more by colleagues who are working in cultural contexts far from those in which the great majority of research in psychology has been, up to now, conducted. We need themes of research that permit us to pass from one culture to another, one religion to another, one spirituality to another.

Pargament and colleagues use the metaphor of the journey for describing the course of the life span: They describe the individual entering the world as « embarking on a religious and spiritual journey that takes him or her on multiple pathways over time » (Pargament et al. 2013, p.5). Having described the hazards of the journey they conclude further on: « The individual’s religious and spiritual pathways say something very important about who that person is; no person’s route in life is identical to that of another. » (Pargament et al. 2013, p.6).

« Who that person is »: it is exactly the question that I have chosen as the theme of reflection for this paper: the construction of the individual identity. My goal was to encourage research in the domain of R/S in the construction of psychological identity, because this topic, as well as that of coping, can play the role of point of convergence between different psychological approaches to religion and spirituality.
